# PD-L1 expression in microvascular endothelial cells predicts the efficacy and side effects of anlotinib

**DOI:** 10.3389/fonc.2025.1544278

**Published:** 2025-07-30

**Authors:** Yingfang Feng, Yuan Gao, Shaochuan Liu, Tingting Qin, Yan Zhang, Jing Wang, Kai Li

**Affiliations:** ^1^ Department of Thoracic Oncology, Tianjin Medical University Cancer Institute & Hospital, Key Laboratory of Cancer Prevention and Therapy, National Clinical Research Center for Cancer, Tianjin’s Clinical Research Center for Cancer, Tianjin, China; ^2^ Department of Radiation Oncology, Tianjin Medical University Cancer Institute & Hospital, Key Laboratory of Cancer Prevention and Therapy, National Clinical Research Center for Cancer, Tianjin’s Clinical Research Center for Cancer, Tianjin, China; ^3^ Department of Oncology, Tianjin Haihe Hospital, Tianjin, China

**Keywords:** anlotinib, PD-L1, microvascular endothelial cells, immune checkpoints, melanoma

## Abstract

**Background:**

Immunotherapy plays a crucial role in the treatment of tumors. However, few studies have investigated the relationship between the expression of Programmed Cell Death Ligand 1 (PD-L1, CD274) in microvascular endothelial cells (MECs), including blood endothelial cells (BECs) and lymphatic endothelial cells (LECs), and immune cell infiltration within tissues.

**Methods:**

In our study, we utilized data from The Cancer Genome Atlas, a mouse subcutaneous xenograft model, and immunofluorescence and immunohistochemical staining to investigate the relationship between PD-L1 expression in melanoma MECs at different tumor stages and the infiltration of CD8^+^ T cells in tumor and normal organs, under conditions with and without anlotinib treatment.

**Results:**

We found that PD-L1 expression was upregulated in tumor MECs, while anlotinib downregulated PD-L1 expression in both tumor and normal tissue MECs, corresponding with increased infiltration of CD8^+^ T cells in the tissues. Additionally, the antitumor effect of anlotinib was most pronounced when administered during the mid-stage of tumor development.

**Conclusions:**

This study evaluated the most effective timing for anlotinib to downregulate PD-L1 expression in tumor and normal tissues to promote immune infiltration. Our findings may offer valuable insights for the clinical use of anlotinib and its potential side effects.

## Introduction

Melanoma, a malignant tumor that develops from pigment cells, has been widely studied and is used as a model for immunotherapy research due to its increased immunogenicity and immune cell infiltration into tumors compared to other cancer types (1).

The tumor microenvironment (TME) contains infiltrating immune cells, fibroblasts, the tumor vascular system, the tumor lymphatic system, and peritumoral cells (2). TME chemokines such as vascular endothelial growth factor (VEGF), fibroblast growth factor (FGF), platelet-derived growth factor (PDGF), and other chemokines stimulate the production of blood endothelial cells (BECs) during the formation of new blood vessels required for cancer growth. Interestingly, tumor BECs also express a variety of immunosuppressive receptors, such as FasL, CD73, VACM-1, and programmed cell death ligand 1 (PD‐L1, CD274), which are involved in impeding the infiltration of immune effector cells in the adjoining tissue around the vessel (3, 4). Similarly, growth factors VEGF-C and VEGF-D produced by the TME and malignant cells can promote lymphagiogenesis and lymphatic remodeling associated with cancer progression (5–7). Lymphatic endothelial cells (LECs) have also been shown to express multiple inhibitory receptors either, including PD-L1 (8), which is involved in peripheral tolerance. Furthermore, it has been found that PD-L1 expression in LECs is regulated by IFN-γ produced by infiltrating lymphocytes, and that PD-L1 directly exerts local inhibitory effects by binding to CD8^+^ T cells in close proximity to the tumor (9). However, the dynamic changes and regulation of PD-L1 on microvascular endothelial cells (MECs), including BECs and LECs, in normal and tumor tissues during tumor growth are not well understood.

Anlotinib is a multi-targeted tyrosine kinase inhibitor that acts on vascular endothelial growth factor receptors 1–3 (VEGFR1–3), fibroblast growth factor receptors 1–4 (FGFR1–4), platelet-derived growth factor receptors α and β (PDGFRα/β), and c-Kit. It inhibits tumor cell proliferation while anti-tumor angiogenesis (10, 11). Due to its efficacy in patients with advanced non-small cell lung cancer (NSCLC), anlotinib monotherapy has been approved by the China Food and Drug Administration in many malignancies (12).

Our previous studies demonstrated that tumors can induce PD-L1 expression in BECs, inhibit the infiltration and activity of CD8^+^ T cells within tumor tissues, and increase the infiltration of FoxP3^+^ T cells, while anlotinib can inhibit tumor growth by downregulating PD-L1 expression in tumor BECs, thereby removing or weakening this immune barrier (13). Additionally, in a recent randomized double-blind trial, we discovered that PD-L1 was expressed in MECs, and that anlotinib enhanced the efficacy of PD-L1 antibody by downregulating PD-L1 expression in MECs, including both BECs and LECs (14). Notably, the impact by anlotinib on PD-L1 in MECs within normal organs—which may lead to the infiltration of immune effector cells and could be associated with immune-related side effects—was not observed in our previous research This suggests that the risk of severe inflammation during anlotinib treatment still need to be further elucidated.

In this study, we continuously observed the dynamic expression of PD-L1 in MECs within tumor and distant normal tissues at different stages of tumor growth. Furthermore, we evaluated the most effective intervention period for anlotinib to downregulate PD-L1 expression in MECs, along with anlotinib’s antitumor effect and its impact on immune cell infiltration in normal tissues, highlighting the need to consider the potential risk of toxic side effects of anlotinib on normal tissues.

## Materials and methods

### Gene expression analysis

We constructed a CD274 mRNA expression map using data from the Human Protein Atlas (HPA) database (https://www.proteinatlas.org/).

The “Gene DE” module of the Tumor Immunity Assessment Resource version 2 (TIMER2) (http://timer.cistrome.org/) was used to analyze differences in CD274 expression between tumor and non-tumor tissues across various tumor types. CD274 expression between primary and metastatic cutaneous melanoma (SKCM) was also evaluated.

### Prognostic analysis of survival

Using TCGA data, we analyzed the correlation between CD274 expression levels and tumor pathologic stage through the UALCAN database (UALCAN (uab.edu)), and generated overall survival (OS) Kaplan–Meier (K–M) plots using the “Survival Analysis” module of Gene Expression Profiling Interactive Analysis Version 2 (GEPIA2) (http://gepia2.cancer-pku.cn/).

### Cell culture and reagents

B16 cells were obtained from the Tianjin Medical University Cancer Institute and Hospital. Anlotinib was provided as a gift by Nanjing Chia Tai Tianqing Company. The cells were cultured in DMEM medium containing 10% fetal bovine serum (FBS) at 37°C in a humidified incubator with 5% CO2.

### *In vivo* experiments

Female C57BL/6J mice aged 6–8 weeks were purchased from SPF (Beijing) Biotechnology Co. Ltd. All experimental procedures were conducted in accordance with protocols approved by the Institutional Animal Care and Research Advisory Committee of Tianjin Medical University. To construct a tumor-bearing mouse model using B16 cells, we subcutaneously injected 1 × 10^6^ B16 cells in 100 μL into the right groin region of each mouse. Tumor growth was monitored daily for several weeks. Based on previous studies, tumor volume was calculated using the formula: volume = 0.52 × length × width × width (15), and day 22 was designated as the study endpoint for mouse survival. According to tumor size (16), tumors were classified into three stages: 5–9 days after implantation as early stage, 10–14 days after implantation as mid stage, and 15–20 days after implantation as late stage. To determine the optimal timing of anlotinib administration, we established a B16 xenograft tumor model and divided the mice into four groups: Control (PBS, 1.5 mg/kg, once daily), An5–9 (anlotinib, 1.5 mg/kg, once daily on days 5–9), An10–15 (anlotinib, 1.5 mg/kg, once daily on days 10–15), and An16–20 (anlotinib, 1.5 mg/kg, once daily on days 16–20). We compared changes in MECs PD-L1 between normal mice and untreated tumor-bearing mice on days 10, 15, and 22. For mice in each treatment group, day 22 after tumor implantation was selected as the observation point; mice in both the control and treatment groups were sacrificed at this time. Tissues were collected for subsequent immunohistochemistry and multicolor immunofluorescence staining.

### Selection of tissue (organs) to observe the PD-L1 on MECs

According to previous research, the characteristics and functions of PD-L1 expressed by BECs and LECs differ, suggesting that they may affect CD8^+^ cells in tissue through different mechanisms. Therefore, BECs and LECs were observed separately. We selected both tumor and normal tissues for observation, as both can be affected by anlotinib. Based on commonly observed immunotherapy-related side effects, we selected ear tissue and kidney tissue to represent peripheral skin and internal organs, respectively.

### Immunofluorescence staining

The fresh frozen sections were rewarmed at room temperature for 10 min, then fixed with 4% paraformaldehyde precooled to 4 °C for 15 min. After fixation, the frozen tissue samples were permeabilized with 0.2% TritonX-100 diluted in PBS for 10 min. The sections were washed with PBS three times for 5 min each, then incubated in PBS blocking solution containing 1% BSA, 0.01% Triton X-100, and 10% FBS at room temperature for 1 h. The slices were incubated with primary antibodies overnight at 4 °C. The primary antibodies used were as follows: rat anti-mouse CD31 (AB56299, Abcam), Syrian hamster anti-mouse Podoplanin (AB11936, Abcam), and rabbit anti-mouse PD-L1 (LS-C746930, LifeSpan). The sections were then rewarmed at room temperature for 30 min and washed in PBS three times for 5 min each. The tissue sections were stained with fluorescent secondary antibodies and incubated at room temperature for 1 h. The following secondary antibodies were used: donkey anti-rat AF488 (A21208, Invitrogen), donkey anti-Syrian hamster AF546 (A21111, Invitrogen), and donkey anti-rabbit AF647 (A31573, Invitrogen) The sections were then washed in PBS for three times. Finally, all the sections were stained with anti-fluorescence quencher containing DAPI. The stained sections were stored at −20 °C, protected from light, and imaged using the Zeiss Imaginer-Z2.

### Evaluation the percentage of PD-L1^+^ MEC in tissues

CD31 (9) and Podoplanin (17) were used to label BECs and LECs, respectively. To assess the percentage of CD31^+^PD-L1^+^ and Podoplanin^+^ PD-L1^+^ in tissues, areas with the most abundant blood and lymphatic vessels were selected under ×10 microscope. Three to five independent fields were then randomly selected using a ×20 objective lens, and the percentage of CD31^+^ PD-L1^+^ cells in all CD31^+^cells and Podoplanin^+^ PD-L1^+^ cells among all Podoplanin^+^ cells was calculated. The average value was taken and used as the final result for each section (13, 18).

### Immunohistochemistry

Paraffin-embedded tissue was sectioned into continuous slices 4 µm thick. The tissue sections were baked overnight at 65°C, followed by deparaffinization and dehydration. Antigen retrieval by high-pressure cooking was performed, along with blocking of endogenous peroxidase activity. The sections were incubated overnight at 4°C with anti-CD8 antibody (ab316778, Abcam). Afterward, a reaction enhancer was applied, and the sections were incubated with a secondary antibody (PV-9002, ZSGB-BIO) at 37°C for 1 h, followed by DAB staining and hematoxylin counterstaining. Images were captured using an optical microscope. The IHC results were evaluated by two professional pathologists. The frequency of positive cells was graded into five levels: 0 (negative), 1 (1%–25%), 2 (26%–50%), 3 (51%–75%), and 4 (76%–100%). The staining intensity was categorized as 0 (negative), 1 (weak), 2 (moderate), and 3 (strong). The IHC score was calculated by multiplying the two values.

### Statistics

Statistical analyses were performed using SPSS v.24 (IBM Corp.), and statistical graphs were generated using GraphPad Prism 8 (GraphPad Software, USA). All measurement data were expressed as mean ± standard deviation. An unpaired t-test was used for comparisons between two groups, and one-way ANOVA was used for comparisons among multiple groups. The Wilcoxon rank-sum test was used for non-normally distributed data. A p <0.05 was considered statistically significant.

## Results

### Expression analysis of PD-L1 in MECs

**Figure 1:**
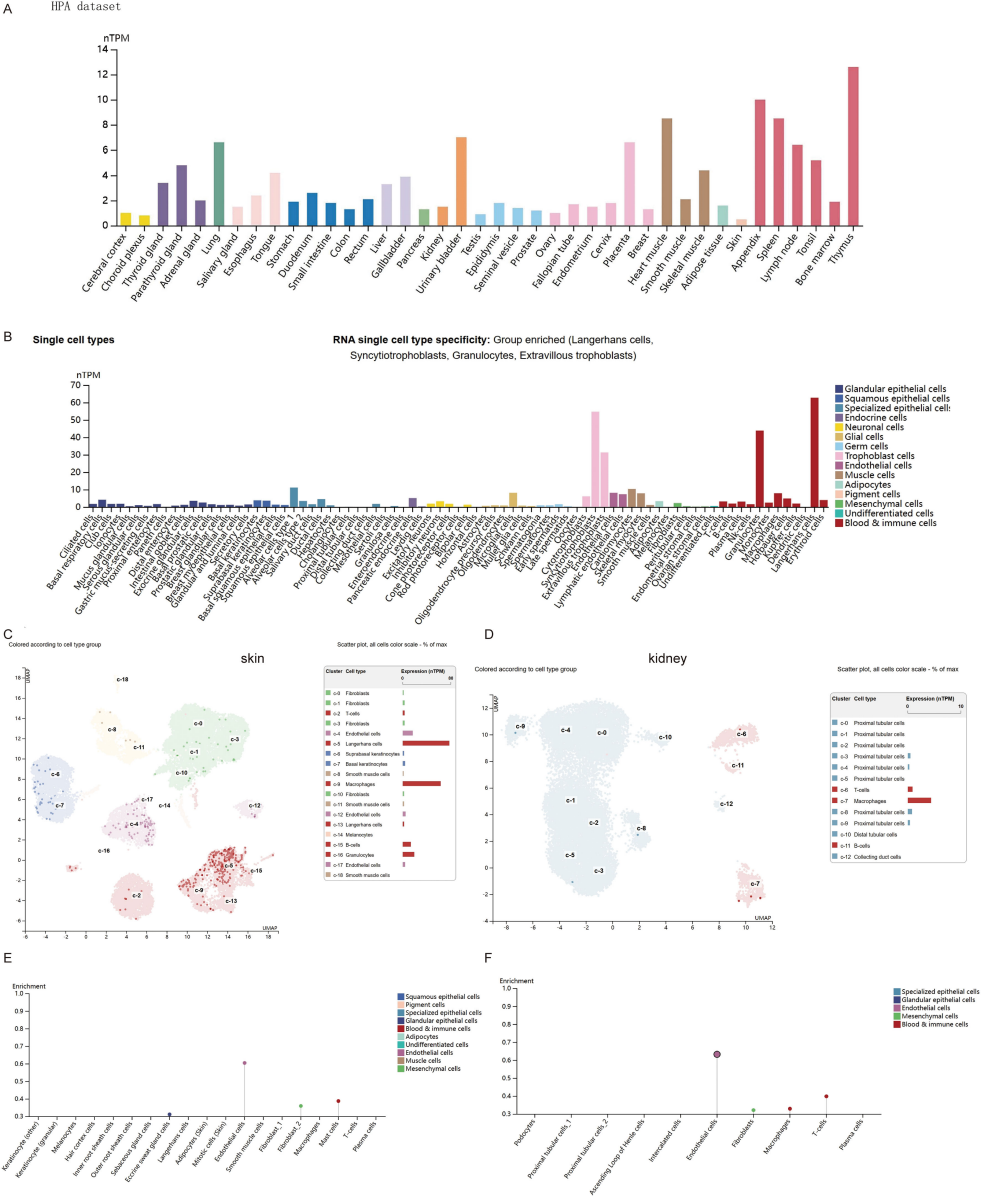
Expression of CD274 in normal tissues and cells. **(A)** CD274 tissue expression based on the Human Protein Atlas (HPA) dataset. **(B)** Expression of CD274 in various single cell types based on the HPA dataset. **(C)** Expression of CD274 in various cell types in the skin based on the HPA dataset. **(D)** Expression of CD274 in various cell types in the kidney based on the HPA dataset. **(E)** Enrichment display of results from **(C)**. **(F)** Enrichment display of results from **(D)**.

In this study, we examined the RNA expression levels of CD274 in various normal human tissues and cells based on the HPA and GTEx databases, and found that CD274 was highly expressed in lymphoid tissues (**Figure 1A**). In addition, CD274 was expressed in a variety of cells, including immune cells, trophoblasts, endothelial cells, and LECs, based on single-cell RNA-seq data (**Figure 1B**). We further selected skin tissue, representing body surface tissue, and kidney tissue, representing internal organs and found that CD274 was highly enriched in the endothelial cells of both tissues.

### PD-L1 expression and prognosis analysis in melanoma

**Figure 2:**
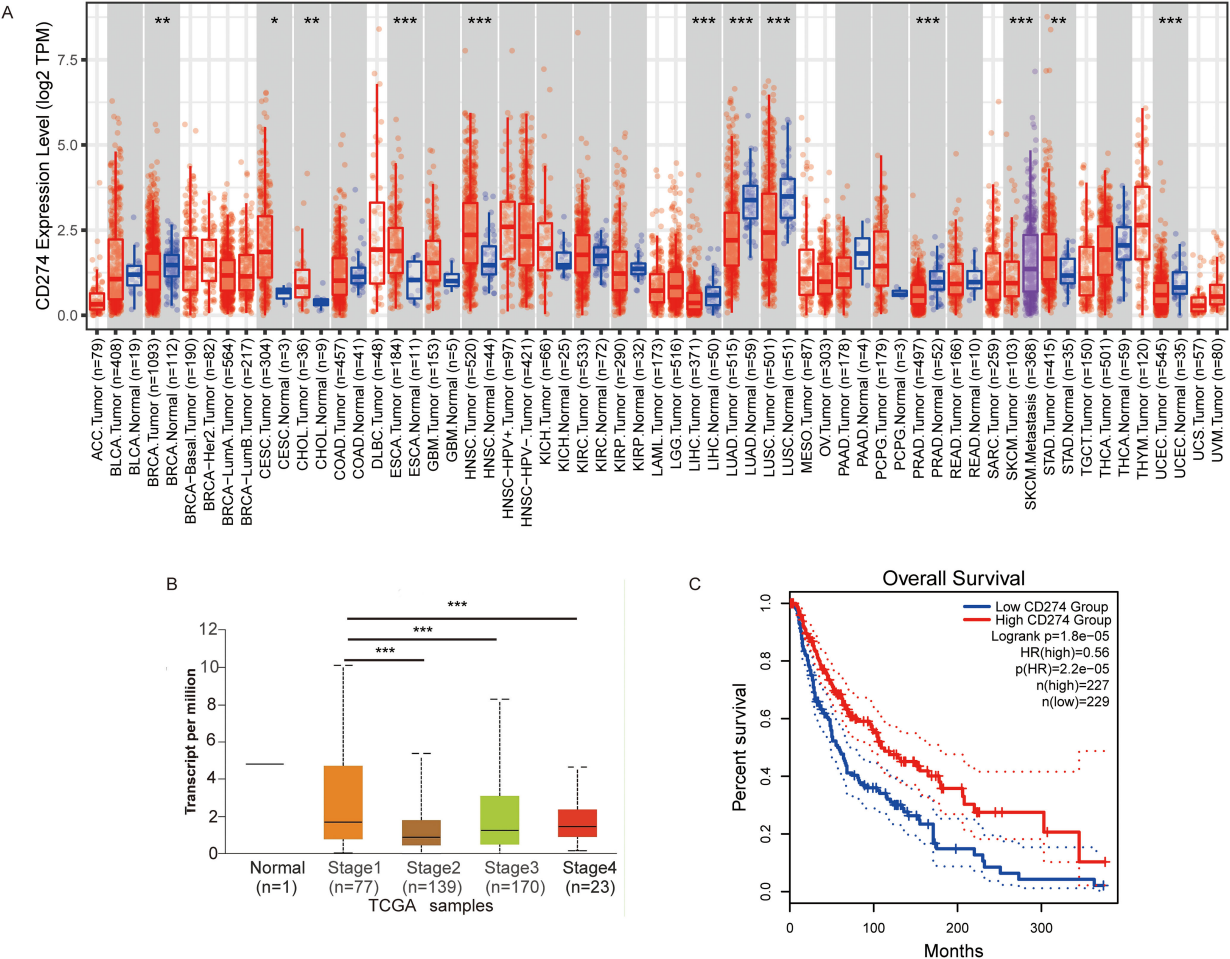
The expression of CD274 in tumor cells is associated with prognosis. **(A)** Visualization of CD274 expression levels in various tumor types using TIMER2. **(B)** Visualization of CD274 expression in different stages of melanoma using the UALCAN database. **(C)** Overall survival analysis of melanoma based on CD274 expression levels constructed using the GEPIA2 database. P-values were determined by log-rank test. *p <0.05; **p <0.01; ***p <0.001.

We next comprehensively evaluated the RNA expression levels of PD-L1 across pan-cancer types using the TCGA database. The expression of PD-L1 differed significantly between most tumors and their corresponding normal tissues. As shown in **Figure 2A**, PD-L1 expression levels were significantly higher than in adjacent normal tissues in the following cancers: cervical squamous cell carcinoma and endocervical adenocarcinoma (CESC), cholangiocarcinoma (CHOL), esophageal carcinoma (ESCA), head and neck squamous cell carcinoma (HNSC), and stomach adenocarcinoma (STAD). PD-L1 expression was also significantly higher in SKCM metastases than in carcinoma *in situ*. Statistical significance, calculated using Wilcoxon’s test, is indicated by the number of asterisks (*: p-value ≤0.05; **: p-value ≤0.01; ***:p-value ≤0.001). In addition, we investigated the correlation between PD-L1 expression and tumor stage using TCGA database, and found that low PD-L1 expression was significantly associated with advanced-stage SKCM (**Figure 2B**). To explore the predictive value of PD-L1 expression level in SKCM, patients were divided into two groups based on PD-L1 levels: high and low expression. OS was significantly longer in patients with high PD-L1 expression compared to those with with low expression (**Figure 2C**). These results suggest that high PD-L1 expression is associated with a more favorable prognosis, consistent with previous studies showing that patients with elevated PD-L1 expression in tumor cells exhibit greater sensitivity to PD-1/PD-L1 inhibitors and achieve improved treatment outcomes (19–23).

### Anlotinib has the best effect on inhibiting tumor growth when administered in the mid stage

**Figure 3:**
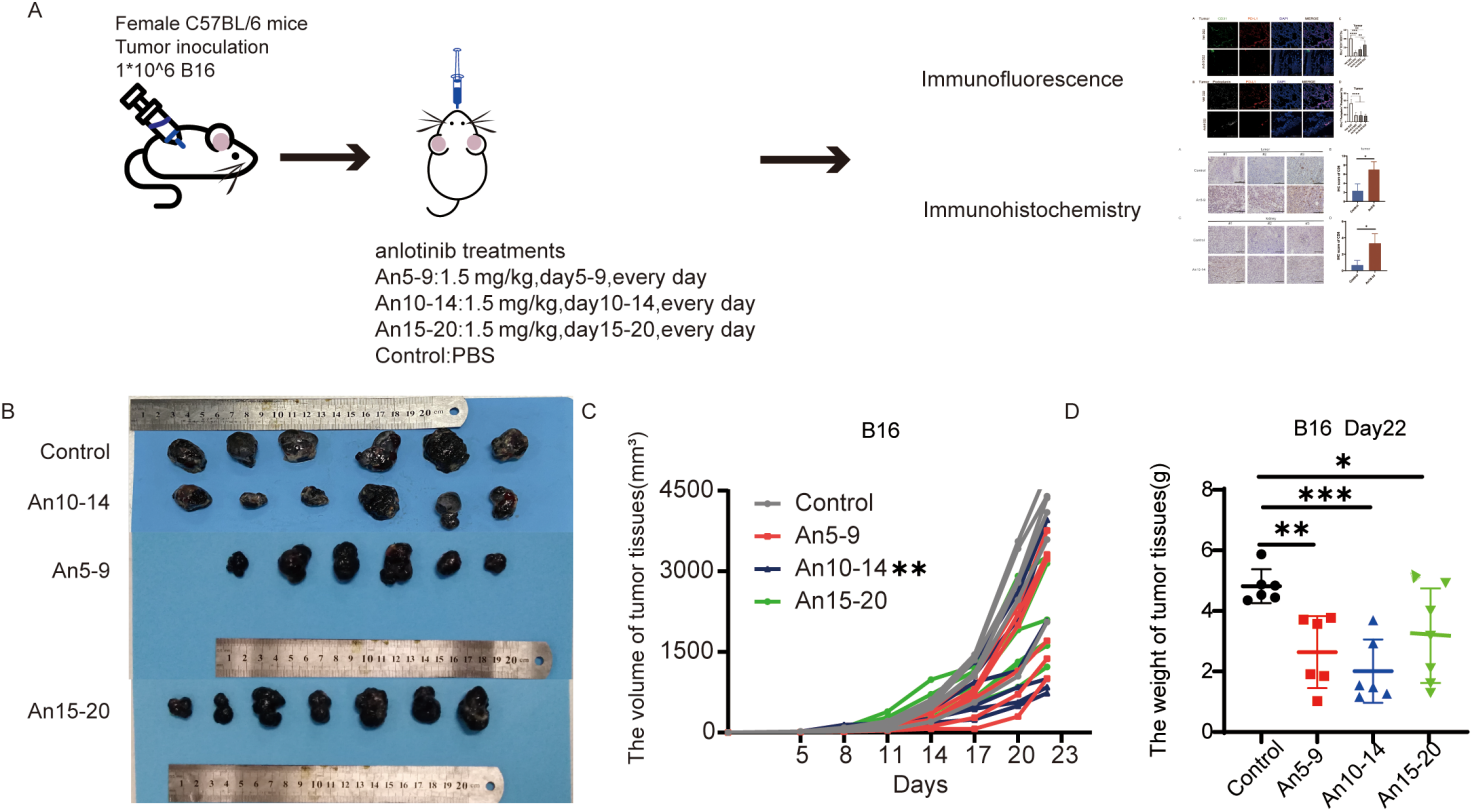
Anlotinib shows the best antitumor effect in the mid-stage of the tumor. **(A)** Schematic of the animal experimental model. C57BL/6 mice were subcutaneously injected with 1 × 10^6^ B16 cells. On day 5, the mice were divided into four groups, with the treatment groups receiving oral administration of anlotinib at 1.5 mg/kg/day from days 5–9, 10–14, and 15–20, while the control group received PBS. **(B)** Representative images of B16 tumors in different groups. **(C)** Tumor growth curves for different treatment groups. **(D)** Weight of B16 tumors in different treatment groups. All data are exhibited as mean ± SD. Statistical differences were assessed using the unpaired Student’s test. *P <0.05, **P <0.01, ***P <0.001.

According to our previous studies, anlotinib reduces tumor volume and weight in the B16 xenograft model (13). To explore whether anlotinib exerts different tumor-inhibitory effects when administered at various stages of tumor progression, we constructed a B16 xenograft tumor model; a schematic diagram is shown in **Figure 3A**. We compared tumor volume and weight on day 22 across four groups and found that tumor growth was inhibited by anlotinib in all three phases, with significantly lower tumor volume in the mid-stage group compared to the early- and late-stage groups (**Figures 3B–D**).

### The expression of PD-L1 in MECs is associated with tumor progression, and can be inhibited by anlotinib

**Figure 4:**
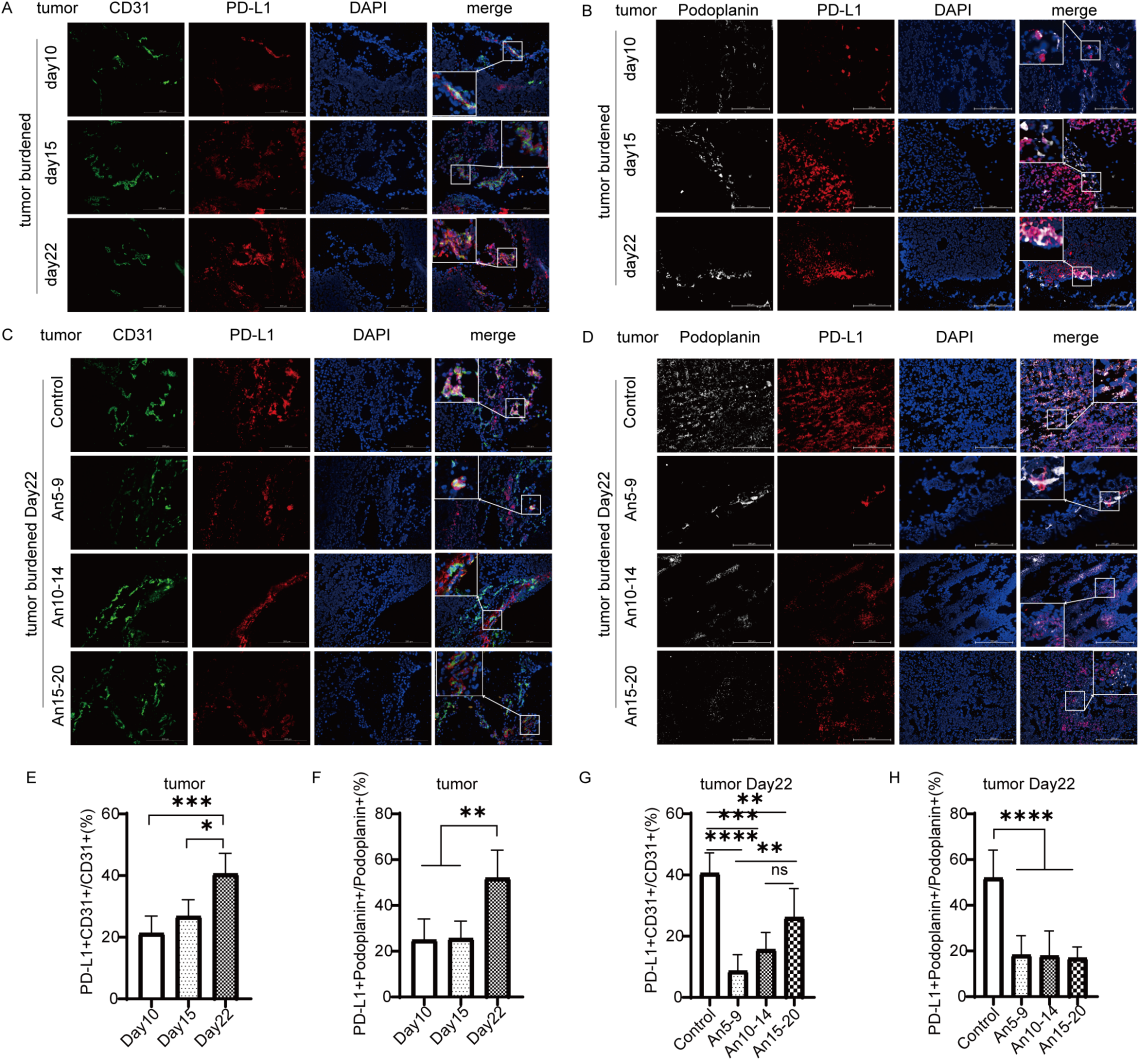
Expression of PD-L1 in tumor microvascular endothelial cells (×200) staining. CD31 in green; PD-L1 in red; Podoplanin in white; DAPI in blue. **(A)** Expression of PD-L1 in tumor BECs on days 10, 15, and 22. **(B)** Expression of PD-L1 in tumor LECs on days 10, 15, and 22. **(C)** Expression of PD-L1 in tumor BECs on day 22 for each treatment group. **(D)** Expression of PD-L1 in tumor LECs on day 22 for each treatment group. **(E)** Statistical graph of PD-L1^+^CD31^+^/CD31^+^ (%) from **(A)**. **(F)** Statistical graph of PD-L1^+^Podoplanin^+^/Podoplanin^+^ (%) from **(B)**. **(G)** Statistical graph of PD-L1^+^CD31^+^/CD31^+^ (%) from **(C)**. **(H)** Statistical graph of PD-L1^+^Podoplanin^+^/Podoplanin^+^ (%) from **(D)**. All data are exhibited as mean ± SD. Statistical differences were assessed using the unpaired Student’s test. *P <0.05, **P <0.01, ***P <0.001, ****P <0.0001.

To further investigate the relationship between PD-L1 expression in MECs and tumor progression, we performed multicolor immunofluorescence staining on tumor tissues. We compared the percentages of PD-L1^+^CD31^+^/CD31^+^ and PD-L1^+^Podoplanin^+^/Podoplanin^+^ cells on days 10, 15, and 22 after tumor cell inoculation. Our results indicated that PD-L1 expression within tumor tissue was upregulated with tumor progression (**Figures 4A, B, E, F**). On day 22 after tumor cell inoculation, MECs exhibited downregulation of PD-L1 expression across all dosing groups compared to the control group (**Figures 4C, D, G, H**), with the most pronounced effect observed in the early treatment group.

### Anlotinib administration in the middle stage of the tumor significantly inhibited PD-L1 expression in LECs of renal tissue

**Figure 5:**
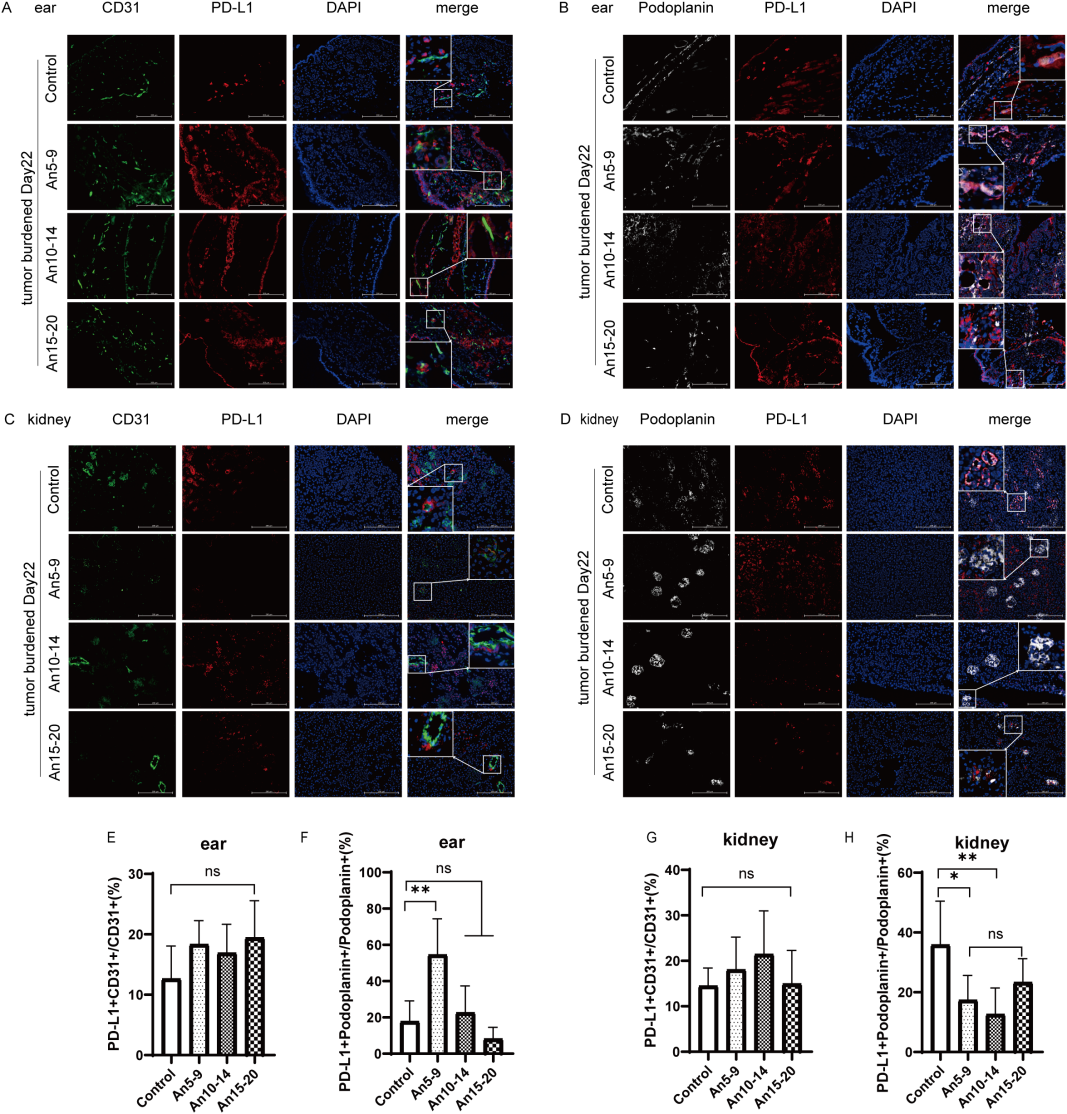
Expression of PD-L1 in normal tissue microvascular endothelial cells (×200) staining. CD31, green; PD-L1, red; Podoplanin, white; DAPI, blue. **(A)** Expression of PD-L1 in BECs from ear tissue on day 22 for each treatment group. **(B)** Expression of PD-L1 in LECs from ear tissue on day 22 for each treatment group. **(C)** Expression of PD-L1 in BECs from kidney tissue on day 22 for each treatment group. **(D)** Expression of PD-L1 in LECs from kidney tissue on day 22 for each treatment group. **(E)** Statistical graph of PD-L1^+^CD31^+^/CD31^+^ (%) from **(A)**. **(F)** Statistical graph of PD-L1^+^ Podoplanin^+^/Podoplanin^+^ (%) from **(B)**. **(G)** Statistical graph of PD-L1^+^CD31^+^/CD31^+^ (%) from **(C)**. **(H)** Statistical graph of PD-L1^+^ Podoplanin^+^/Podoplanin^+^ (%) from **(D)**. All data are exhibited as mean ± SD. Statistical differences were assessed using the unpaired Student’s test. *P <0.05, **P <0.01, ns, no significance.

It has been reported that PD-L1 expression in LECs within normal tissue is more responsive to the local immune microenvironment of tumors, whereas PD-L1 expression in BECs is weakly associated with local tumor lesions (9). Next, we investigated whether anlotinib affected PD-L1 expression in MECs from normal tissues. We selected ear tissue to represent body surface skin and kidney tissue to represent internal organs for multicolor immunofluorescence staining. In ear tissue, compared to the control group, there were no significant differences in PD-L1 expression in BECs among the different dosing groups (**Figures 5A, E**), whereas PD-L1 expression in LECs was upregulated in early- and mid-term dosing groups and downregulated in the late-term group (**Figures 5B, F**). In kidney tissue, compared to the control group, there were also no significant differences in PD-L1 expression in BECs among the different dosing groups (**Figures 5C, G**); however, PD-L1 expression in LECs was downregulated across all dosing groups, with the most pronounced decrease observed in the mid-stage group (An10–14) (**Figures 5D, H**).

### Anlotinib promotes the infiltration of CD8^+^ T cells by decreasing the expression of PD-L1 in microvascular endothelial cells

**Figure 6:**
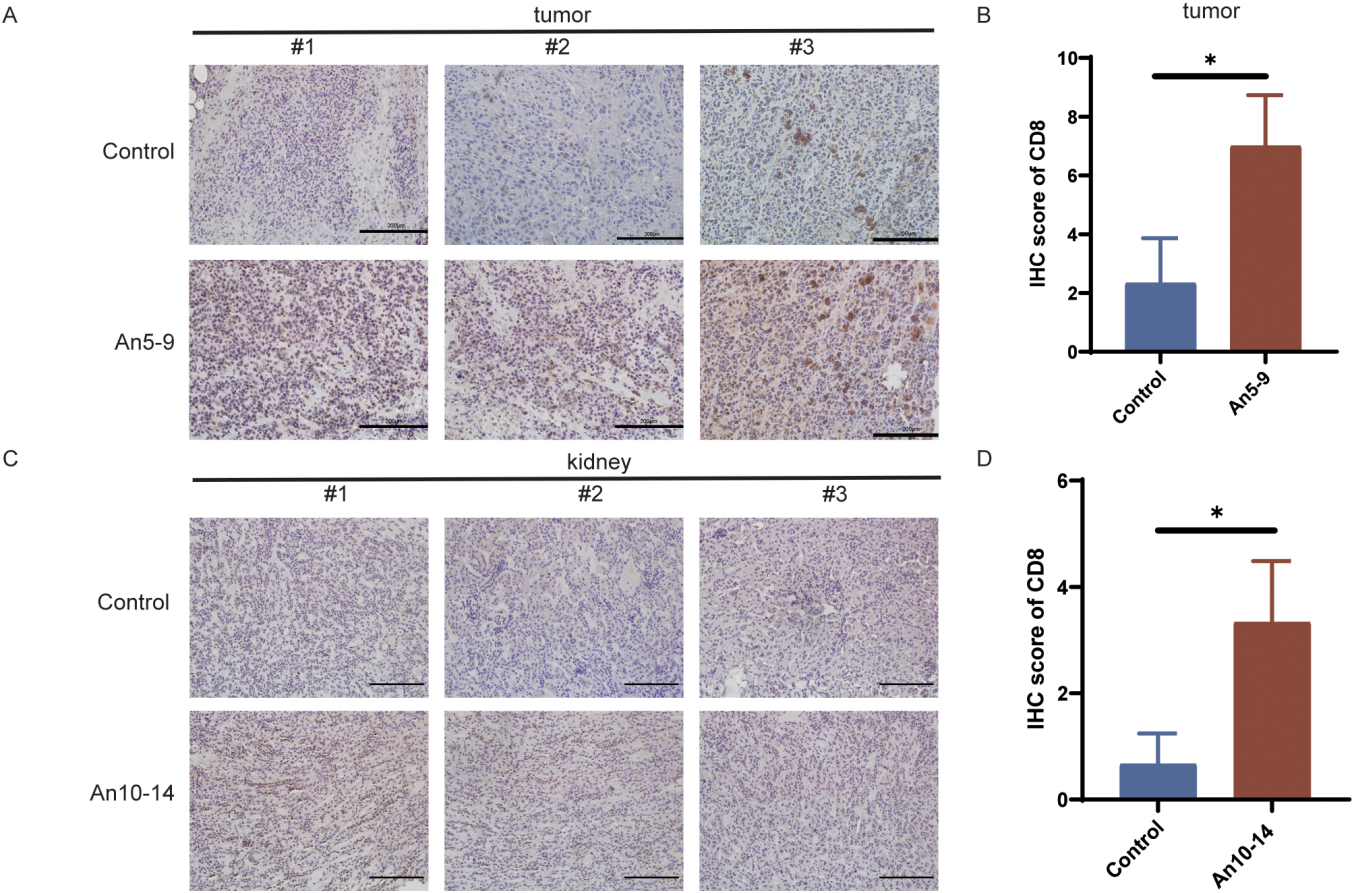
Infiltration of CD8^+^ T cells in different tissues. **(A)** IHC staining of CD8^+^ T cells in tumor tissues from the control group and early treatment group. Scale bar, 200 µm. **(B)** CD8 IHC staining scores in tumor tissues from the control group and early treatment group. **(C)** IHC staining of CD8^+^ T cells in kidney tissues from the control group and mid-treatment group. Scale bar, 200 µm. **(D)** CD8 IHC staining scores in kidney tissues from the control group and mid-treatment group. Data are presented as mean ± SD, *p <0.05, Student’s t-test.

The results indicate that anlotinib reduces PD-L1 expression in MECs within tumor tissues (**Figures 4C, D, G, H**) and downregulates PD-L1 expression in LECs within kidney tissues (**Figures 5D, H**). To assess the effect of anlotinib administration at different stages on CD8^+^ T cell infiltration in tumor tissues, we performed immunohistochemical staining, which showed increased CD8^+^ T cell infiltration in the early-stage dosing group (An5–9) compared to the control group (**Figures 6A, B**). Furthermore, to evaluate the impact of anlotinib administration at different stages on CD8^+^ T cells in kidney tissues, immunohistochemical analysis revealed increased CD8^+^ T cell infiltration in the mid-stage dosing group compared to the control group (**Figures 6C, D**), suggesting that anlotinib may promote inflammation in the kidneys. These findings suggest that PD-L1 expression in MECs restricts CD8^+^ T cell infiltration, while anlotinib inhibits PD-L1 expression in MECs, thereby promoting CD8^+^ T cell infiltration in tissues.

## Discussion

The application of immune checkpoint inhibitors (ICIs) has significantly transformed cancer treatment. ICIs targeting PD-L1 have demonstrated notable antitumor efficacy in various cancers. However, only a subset of patients benefits from clinical application (24). This may be partly due to PD-L1 expression by MECs in the tumor microenvironment, which can influence the infiltration and function of CD8^+^ T cells.

Lane et al. (9) found that BECs and LECs can express PD-L1 in tumor tissues and inflamed skin. Our recent studies (13, 14) indicate that PD-L1 is highly expressed in BECs and LECs within tumor tissues. This study, based on the HPA dataset, further confirms high PD-L1 expression in endothelial cells at the transcriptional level (**Figure 1B**). Additionally, endothelial cells in renal and skin tissues also exhibit high PD-L1 expression (**Figures 1C–F**). Based on these results, this study first explores the relationship between PD-L1 expression and tumor progression, finding that PD-L1 expression in tumor-associated MECs is upregulated during tumor progression (**Figures 4A, B, E, F**). PD-L1 in BECs can be induced by the following pathways: the hypoxia-HIF-1α-PD-L1 pathway (25), the VEGFR-AKT-PD-L1 pathway (13, 26), and the IFN-γ-JAK/STAT-PD-L1 pathway (27, 28). LECs can act as antigen-presenting cells, actively clearing exogenous antigens and cross-presenting them to homologous CD8^+^ T cells. This interaction results in elevated PD-L1 expression in LECs (8, 29). Moreover, infiltrating CD8^+^ T cells in the tissue are located near lymphatic vessels, and their secretion of IFN-γ can induce PD-L1 expression in LECs (9, 30). Potential mechanisms underlying PD-L1 modulation in MECs may involve VEGFR2/PI3K/AKT pathway inhibition, as demonstrated in intrahepatic cholangiocarcinoma (31).

PD-L1 expression in BECs restricts CD8^+^ T cell infiltration and activity and enhances Treg cell infiltration by binding to PD-1 on T cells within the vasculature (13, 14). Unlike BECs, CD8^+^ T cells are in close proximity to the lymphatic vessel system within tumors; thus, PD-L1 in LECs exerts ‘local inhibitory effects’ by binding to CD8^+^ T cells in adjacent tumor tissue (8, 9, 29), mediating apoptosis of tumor-specific CD8^+^ TCM cells and promoting to immune evasion (32). Anlotinib can increase CD8^+^ T cell infiltration and activity in tumor tissue by downregulating PD-L1 in BECs through inhibition of the VEGFR-AKT signaling pathway (13). Furthermore, we have confirmed that anlotinib can inhibit VEGFR signaling (15), which is involved in regulating PD-L1 expression in LECs (33); thus, anlotinib may enhance the efficacy of PD-L1 antibodies by downregulating PD-L1 in LECs in clinical settings (14). However, there no studies have explored the optimal timing for anlotinib administration; therefore, we treated tumor-bearing mice with anlotinib at three distinct stages of tumor progression. The results indicated that administering anlotinib during the mid-stage of tumor growth yielded the most anti-tumor effects (**Figures 3C, D**). In contrast, early treatment of anlotinib resulted in the most significant downregulation of PD-L1 expression on MECs (**Figures 4C, D, G, H**). Additionally, immunohistochemical results showed an increase in infiltrating CD8^+^ T cells in tumor tissue in the early treatment group compared to the control group (**Figures 6A, B**). This is consistent with our previous findings (13). Recent research has demonstrated that the combination of anlotinib and murine CIK cells enhances the expression of granzyme B and interferon-γ in CD8^+^ T cells (34). Additionally, anlotinib is capable of inducing CCL5-mediated recruitment of CD8^+^ T cells (35). Recent investigations have also revealed that anlotinib exhibits similarities in promoting T cell infiltration via transferrin receptor-dependent mechanisms (36). Furthermore, in a phase II single-arm study, anlotinib was found to increase the proportion of NK cells in the plasma of patients with advanced hepatocellular carcinoma (37). In a mouse colorectal cancer model, anlotinib enhanced the proportion of NK cells and M1 macrophages while reducing M2 macrophages in the TME (38). Similarly, in a syngeneic lung cancer mouse model, anlotinib increased the infiltration of innate immune cells, including natural killer (NK) cells and antigen-presenting cells (APCs), such as M1-like tumor-associated macrophages (TAMs) and dendritic cells (DCs), while significantly decreasing the percentage of M2-like TAMs (39). In perioperative NSCLC patients, complete response (CR) cases treated with anlotinib exhibited a reduction in VEGF^+^ cells and CD4^+^FoxP3^+^ Treg cells in the TME, along with an increase in perivascular CD4^+^ T cells, CD39^+^CD8^+^ T cells, and M1 macrophages (40). In a lung adenocarcinoma model, anlotinib promoted apoptosis of cancer-associated fibroblasts (CAFs) and increased tumor-infiltrating CD8^+^ T cells (41). Moreover, in an osimertinib-resistant xenograft model, combination therapy with anlotinib and osimertinib significantly increased CD8^+^ T cell infiltration compared to osimertinib monotherapy (42). These results indicate that anlotinib modulates various immune cell populations within the TME, thereby reversing tumor immune evasion.

Our team previously found, through a randomized double-blind trial, that downregulation of PD-L1 in the tumor microvascular endothelium led to synergistic effects when anti-PD-L1 antibody was combined with anlotinib (14). The above results imply that anlotinib has distinct effects: when used as monotherapy, mid-stage treatment yields the greatest efficacy through its direct anti-tumor and antiangiogenic function on pronounced angiogenesis during the mid-stage of tumor growth. These findings also provide a theoretical basis for combining anlotinib with PD-L1 antibodies in the early stage of tumor development, likely through its ‘slow and continuous effect’ downregulation of PD-L1 in MECs.

PD-L1 is expressed in normal tissue microvascular endothelial cells, and inflammatory processes can induce the expression of PD-L1 in peripheral LECs (43). Here, we also focus on whether anlotinib might cause immune-related inflammation in normal tissues. Therefore, we selected two tissue types representing surface and visceral organs—specifically, skin and kidney tissues. We found that anlotinib did not significantly affect PD-L1 expression in BECs of either normal tissues. However, PD-L1 levels in LECs from both normal tissues were notably downregulated after anlotinib treatment, although the degree of downregulation in ear tissue was only marginally statistically significant. These results provide the first evidence that normal tissues can respond to anlotinib, as it exerts similar effects on PD-L1 in MECs within tumors. This suggests that the risk of immune-related side effects may arise during treatment, especially when anlotinib is used in combination with PD-L1 antibodies. To confirm this hypothesis, we performed immunohistochemical staining of kidney tissue, where we observed a substantial increase in infiltrating CD8^+^ T cells following anlotinib treatment compared to the control group (**Figures 6C, D**). This highlights the need for great caution regarding potential adverse effects on the kidney.

This study has several limitations. The analysis of normal tissues was limited to the ear and kidney; expanding this to other organs, such as the heart and lung, would provide a better assessment of systemic immune-related toxicity. The mechanisms by which PD-L1 expression in tumor-associated microvascular endothelial cells influences the tumor immune microenvironment remain to be fully explored, requiring further experimental work to clarify these interactions. Although the B16 xenograft model offers a controlled system, patient-derived xenograft models or 3D organoid models may better simulate human tumor-immune interactions. The reasons for the differential responses to anlotinib treatment in PD-L1 expression in BECs and LECs of the ear and kidney still require further investigation. Long-term studies on the effects of anlotinib on PD-L1 downregulation and immune infiltration are necessary to balance therapeutic efficacy and chronic toxicity.

### Conclusion

In conclusion, this study demonstrates that PD-L1 expression in tumor-associated microvascular endothelial cells is related to tumor staging and identifies the optimal timing for anlotinib to enhance immune cell infiltration in tumors, confirming the findings of our previous research. Additionally, this is the first study to illustrate the optimal timing for anlotinib administration to downregulate PD-L1 in MECs. Moreover, our results are the first to demonstrate that anlotinib can also downregulate PD-L1 in MECs and enhance CD8^+^ cell infiltration in normal tissues. We propose screening tumor types that exhibit greater sensitivity to anlotinib in combination with immunotherapy, based on PD-L1 expression levels in MECs. In addition to the solid tumors already investigated, the potential of combining anlotinib with immunotherapy should also be assessed in other tumor types characterized by high PD-L1 expression.

Consequently, our findings provide a theoretical basis for the clinical application of anlotinib in combination with immunotherapy and highlight the need for caution regarding immune-related inflammation in normal organs.

## Data Availability

The raw data supporting the conclusions of this article will be made available by the authors, without undue reservation.
